# Evaluating the implementation of adult smoking cessation programs in community settings: a scoping review

**DOI:** 10.3389/fpubh.2024.1495151

**Published:** 2025-03-28

**Authors:** Remai Mitchell, Kerry-Ann F. O’Grady, David Brain, Megumi Lim, Natalia Gonzalez Bohorquez, Ureni Halahakone, Simone Braithwaite, Joanne Isbel, Shelley Peardon-Freeman, Madonna Kennedy, Zephanie Tyack

**Affiliations:** ^1^Australian Centre for Health Services Innovation (AusHSI), School of Public Health and Social Work Centre for Healthcare Transformation, Queensland University of Technology (QUT), Brisbane, QLD, Australia; ^2^Queensland Public Health and Scientific Services Division, Queensland Department of Health, Brisbane, QLD, Australia; ^3^Health Contact Centre, Queensland Ambulance Service, Queensland Department of Health, Brisbane, QLD, Australia

**Keywords:** smoking cessation interventions, implementation science, theories, models, and frameworks, implementation strategies, scoping review, co-design

## Abstract

**Introduction:**

Tobacco smoking is a leading contributor to preventable morbidity and premature mortality globally. Although evidence-based smoking cessation programs have been implemented, there is limited evidence on the application of theories, models, and frameworks (TMFs), and implementation strategies to support such programs. This scoping review mapped the evidence for interventions, TMFs, and implementation strategies used for smoking cessation programs in the community.

**Methods:**

We searched four electronic databases in addition to grey literature and conducted hand-searching between February and December 2023. Original studies of qualitative, quantitative, or mixed methods were considered for inclusion. Studies reporting prospectively planned and/or delivered implementation of smoking cessation interventions or programs, incorporating contextual factors, use of implementation TMF, implementation strategies, or other factors influencing implementation were considered for inclusion. Intervention components were categorized using the Template for Intervention Description and Replication (TIDieR) checklist. Implementation strategies were mapped to the Expert Recommendations for Implementing Change (ERIC) Strategy Clusters.

**Results:**

A total of 31 studies were included. We identified 12 discrete interventions, commonly included as part of multicomponent interventions. Most studies reported tailoring or modifying interventions at the population or individual level. We identified 19 distinct implementation TMFs used to prospectively guide or evaluate implementation in 26 out of 31 included studies. Studies reported diverse implementation strategies. Three studies embedded culturally appropriate TMFs or local cultural guidance into the implementation process. These studies took a collaborative approach with the communities through partnership, participation, cultural tailoring, and community-directed implementation.

**Discussion:**

Our findings highlight the methods by which the implementation of smoking cessation may be supported within the community. Whilst there is debate surrounding their necessity, there are practical benefits to applying TMFs for implementing, evaluating, and disseminating findings. We determined that whilst ERIC was well-suited as a framework for guiding the implementation of future smoking cessation programs, there was inconsistent use of implementation strategies across the ERIC domains. Our findings highlight a lack of harmonization in the literature to culturally tailor implementation processes for local communities.

## 1 Introduction

Tobacco smoking contributes substantial global health burden through disability-adjusted life years (1), poor health-related quality of life (2), and preventable morbidity and premature mortality (3). In some settings, smoking causes more disease and death than alcohol and illicit drugs combined (4). Whilst overall smoking prevalence has declined in most Organization for Economic Co-operation and Development nations in the past decade (5), morbidity and mortality related to tobacco smoking continues to rise with global population growth (6). Whilst smoking cessation programs such as Quitline have been established as an effective, and cost-effective means of reducing tobacco smoking internationally (7–16), evidence-based interventions and practices may not achieve their full or desired effect if poorly implemented (17, 18).

Implementation science bridges the gap between knowledge and practice by evaluating how interventions that have been shown to be effective at small scale, within the controlled research environment, can be embedded on a large-scale into routine service structure and delivery (19–22). Implementation strategies describe the methods used to implement evidence-based practices into routine service provision, and are essential to ensuring successful implementation (23). However, implementation strategies are infrequently used and reported, or may lack an implementation theory, model, and/or framework (TMF) to support their use (23). Whilst there are a number of implementation TMFs reported in the literature (for example (24–28)), they are rarely applied to prospectively guide the study design, development and conduct, or to support interpretation of results from research projects or implementation studies (29). Limited use of implementation TMFs may be due to lack of provider familiarity or experience, or uncertainty about how to apply TMFs to an implementation effort (30). Reporting implementation strategies alongside TMFs supports the evaluation of how interventions, treatments, and services can be implemented successfully into routine care, at scale (31, 32). Furthermore, prospective use of TMFs can guide the design of implementation strategies (33). Reasons for inconsistent use and reporting of implementation strategies are not well understood, however may be due to confusing definitions, inconsistent application of terminology, and poorly described strategies (23). Proctor (23) argues that the degree of implementation success cannot be evaluated, nor can the implementation effort be replicated, without clear, accurate, and complete reporting of the implementation strategies used.

The implementation of smoking cessation programs has been evaluated previously in a number of systematic and scoping reviews (34–40). Existing reviews have focused on specific contexts, for example, within oncology clinics (34), or in hospitals (35, 36). Other reviews have focused on service provider outcomes (36, 39, 40), implementation outcomes (35, 37, 38), or barriers and facilitators of implementation (36, 40). Based on preliminary searches as part of our study, no reviews of the implementation of smoking cessation programs with a focus on the use of TMFs alongside implementation strategies were identified internationally. Therefore, this review focusses on the use of implementation strategies, guided by TMFs or principles where relevant, in the implementation of smoking cessation programs.

## 2 Objectives

The aim of this scoping review was to map evidence regarding the real-world implementation of smoking cessation programs for adults in community contexts. The study objectives were to evaluate the following:

- What interventions are used in the implementation of smoking cessation programs to facilitate quit success?
- What implementation theories, models, and frameworks are used to guide the implementation of smoking cessation programs?
- What implementation strategies are used for smoking cessation programs?

## 3 Methods

**Figure 1:**
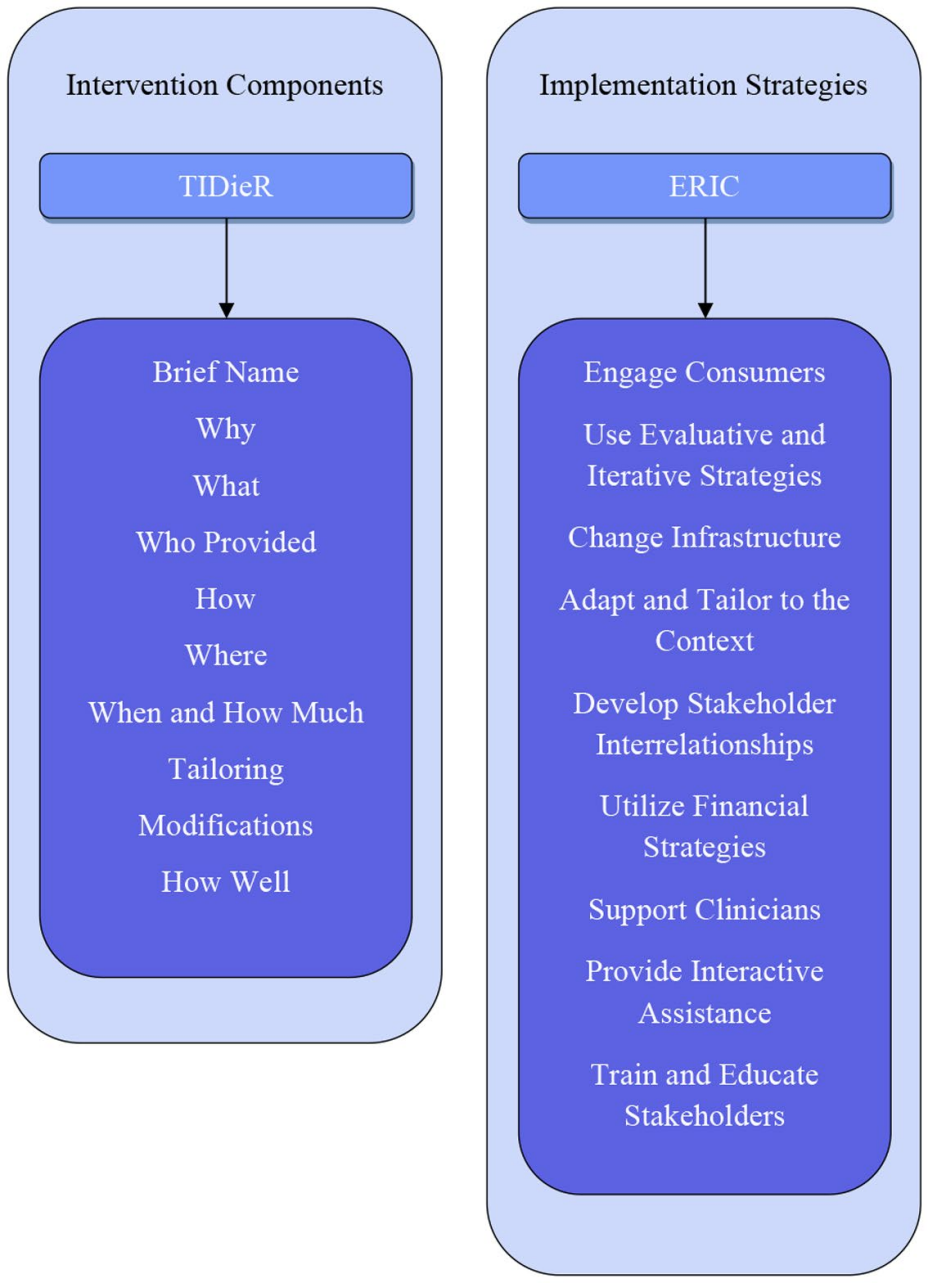
Findings and frameworks for reporting implementation.

A scoping review was selected to map the current literature and enable synthesis of key concepts across a broad range of study designs and topics (41). Scoping reviews are particularly relevant when the area of research is nascent, unclear, or complex (42, 43). Nicotine addiction is particularly complex due to the physiological, psychological, and behavioral drivers of addiction contributing to high prevalence of tobacco use in groups experiencing socioeconomic disadvantage (44, 45). The study protocol provides a full description of the methods, and is available as a pre-print (46). Minor deviations from the protocol included: (1) changes to the research questions in the study objectives to provide greater clarity for the reader; (2) updates to the inclusion and exclusion criteria to ensure potentially relevant papers were included in this review (Tables 1, 2; Figure 1; 46).

**Table 1:** Inclusion criteria.

JBI Population Concept Context mnemonic for scoping reviews used to report the implementation of smoking cessation programs	
PCC element	Inclusion criteria
Population	Adult daily smokers aged 18 years or older
Concepts	Smoking cessation interventions
	Report on the use of implementation strategies for guiding, assessing, or evaluating smoking cessation programs applied prospectively
	Contextual factors
Context	Community-based smoking cessation programs

**Table 2:** Exclusion criteria.

Exclusion criteria
Interventions where smoking cessation was not targeted, for example tobacco use reduction or motivation to quit
Studies that did not directly evaluate determinants of implementation such as barriers and facilitators of implementation, implementation strategies or processes. For example, an implementation evaluation was reported but alignment with our criteria was unclear due to lack of detail, and/ or use of an implementation TMF (53, 140, 141)
Reporting exclusively on theoretical or conceptual research, or the use of an implementation TMF to retrospectively guide evaluation rather than prospective use
Studies reporting exclusively on clinical or patient-reported outcomes, i.e., no implementation was reported according to our criteria
Studies reporting exclusively on service provider outcomes, i.e., service user outcomes were not directly evaluated

### 3.1 Identifying relevant studies

We searched Medline via EBSCOhost, Cochrane CENTRAL, Embase, and Web of Science in addition to grey literature and hand searching for published and unpublished studies. Database searching took place on 21 February 2023, and grey literature searching took place from 21 to 23 August 2023. We performed forward and backward citation searching for all included studies via Citation Chaser (47) on 06 December 2023. Original qualitative, quantitative or mixed methods studies conducted between June 1997 and the final search date were considered for inclusion, consistent with the date of inception of the first Quitline in the world in Victoria, Australia (48). No language restrictions were used. The search strategy was adapted for each database or information source in consultation with a research librarian (Supplementary Table 1). Citations identified and retrieved from the search were loaded into EndNote 20.0.12021 (Clarivate Analytics, PA, USA) citation management system, and duplicates were removed. Titles and abstracts of remaining articles were loaded into the online platform, Rayyan (49).

### 3.2 Study selection

The criteria for included studies were based on the JBI Population Concept Context (PCC) mnemonic for scoping reviews (50). Studies reporting a smoking cessation intervention or program in which implementation was prospectively planned and/or delivered and broadly incorporated one or more of the following: (1) implementation strategies; (2) use of a TMF or other factors that influenced planning and delivery of implementation as defined by the authors; (3) contextual factors influencing implementation. This review focusses on implementation strategies and the TMFs guiding these strategies. Contextual factors are beyond the scope of this review and will be reported in a separate paper. The inclusion criteria for studies are described in Table 1, and exclusion criteria described in Table 2.

**Figure 2:**
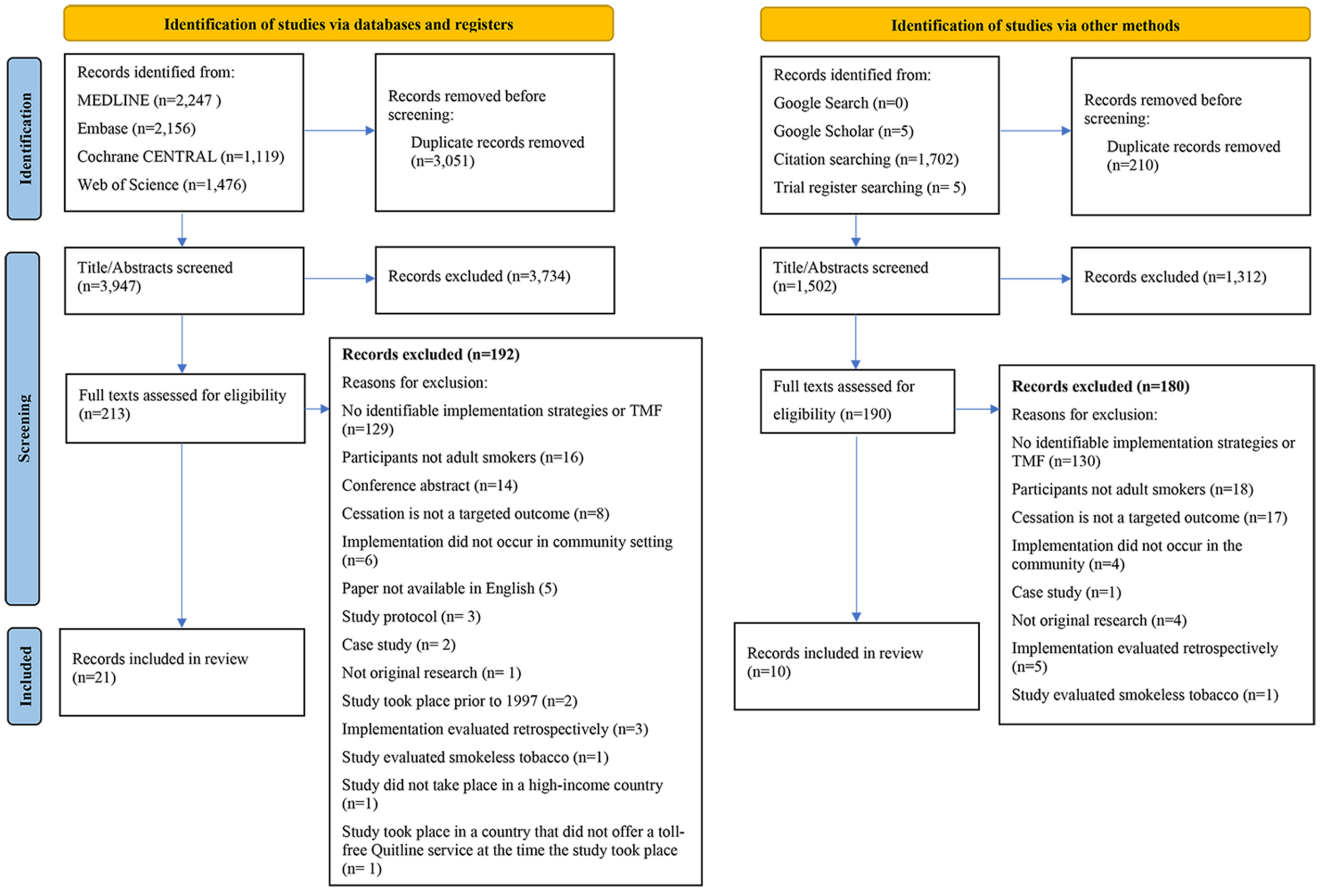
PRISMA-ScR diagram.

Title and abstract screening of all studies retrieved during the search was performed independently by three authors; one author (RM) screened all articles, and the remaining two authors (NGB, ML) divided the number of articles evenly to screen for potential inclusion. Conflicts that arose during the screening process were resolved by an independent third reviewer; one author (NGB) resolved conflicts between RM and ML, while ML resolved conflicts between RM and NGB. Where conflicts were not able to be resolved, an additional reviewer resolved the conflicts (ZT). Full-text screening of all articles that passed title and abstract screening was performed by one author (RM) for eligibility against the inclusion criteria, with a random 20 percent verified by ZT. Conflicts that arose during full-text screening were resolved by KOG and DB. Results of the search and the process of inclusion for studies, including reasons for exclusion of studies that underwent full-text screening was reported in the PRISMA extension for scoping reviews (PRISMA-ScR) (51) (Figure 2).

### 3.3 Charting the data

Data were extracted from papers by two reviewers (RM, UH) using the data extraction template developed by the authors, and relevant to the review questions (Supplementary Table 2). The data extraction template was pilot tested and refined by RM and ZT using five randomly included citations, prior to formal extraction of all included papers. Twenty percent of the data extraction from all included studies was validated by two independent reviewers (NGB and ML). Study identifiers, for example, author, year of publication, country, study setting, participant population, sample size and study design were extracted. Data were categorized according to predetermined categories including intervention of interest, implementation TMFs, and implementation strategies (52). Implementation strategies reported in included papers were mapped according to the discrete strategy clusters within the nine domains described in The Expert Recommendations for Implementing Change (ERIC) (53).The ERIC clusters are a categorization of 73 implementation strategies, organized as a guide to implementers for selecting the most appropriate strategies specific to their context, to support the implementation effort (53). Extraction and charting of the data was an iterative process, verified through discussion by four authors (RM, ZT, KOG, DB) until consensus was reached.

### 3.4 Collating, summarizing, and reporting the results

Quantitative and qualitative data from all included studies were extracted and reported using graphical and tabular descriptions of the results, and via narrative synthesis where appropriate. Intervention components were categorized using the template for intervention description and replication (TIDieR) checklist (54). Implementation TMFs as stated by the study authors were extracted and collated into a spreadsheet. We further categorized named TMFs within each study, or other identifiable implementation processes where no TMF were reported (Table 3). We checked for evidence on how the chosen TMFs were applied, and their alignment with planning, implementation and evaluation of outcomes. Implementation strategies used in studies were extracted and synthesized according to the nine ERIC strategy domains (53). We also aimed to include implementation strategies that fell outside of the given frameworks and report them separately. Implementation outcomes, and contextual factors and processes evaluated in included studies will be reported separately as they are beyond the scope of this paper.

**Table 3:** Implementation TMFs, or other approaches used to prospectively guide implementation or evaluation.

First author, year	Implementation TMF
Hayes, 2022	AIM-IAM-FIM
Gould, 2019	BCW
Little, 2009	Bracht’s Five Stage Community Organization Model
Andrews, 2011	CBPR framework
Meijer, 2021	CFIR
Tong, 2023	ERIC
Landais, 2021	IM Adapt
Foley, 2023; Scheffers-van Schayck, 2021	Implementation-effectiveness hybrid design
Darker, 2022	MRC
Jones, 2020	NPT
Abdelmutti, 2019	OMRU
Windsor, 2014; Windsor, 2017	PEM
Matthews, 2009	PEN-3
De Los Reyes, 2023	PRECEDE-PROCEED
Blok, 2019; Japuntich, 2022	PRISM
Blok, 2019; Craig, 2022; Hood-Medland, 2019; Kim, 2012; Shorey Fennell, 2023; Vidrine, 2013; Wetter, 2007	RE-AIM
Jones, 2020; Gould, 2019; Ni Mhurchu, 2019	TDF
Skelton, 2022	TFA
Meijer, 2021	UTAUT
First author, year	Other approaches
Fullerton, 2015	Collaborative participatory approach
Gould, 2019	Co-design with Aboriginal Medical Services
Hayes, 2022	Community-based participatory approach
Lachter, 2022	Community engagement framework
Ni Mhurchu, 2019	Co-design with Māori and Pasifika communities

AIM-IAM-FIM, Acceptability of Intervention Measure; Intervention Appropriateness Measure; and Feasibility of Intervention Measure; BCW, Behavior Change Wheel; CBPR, Community Based Participatory Research framework; CFIR, Consolidated Framework for Implementation Research; ERIC, Expert Recommendations for Implementing Change; IM Adapt, Intervention Mapping for Adaptation; MRC, Medical Research Council framework for design and evaluation of complex interventions to improve health; NPT, Normalization Process Theory; OMRU, Ottawa Model of Research Use knowledge translation framework; PEM, Process Evaluation Model; PEN-3, Impact of a behavior on health (Positive, Existential or Negative); influences of the behavior (Perceptions, Enablers, or Nurturers); focus of the health behavior intervention (Person, Extended family, or Neighborhood); PRISM, The Practical, Robust Implementation and Sustainability Model; RE-AIM, Reach, Effectiveness, Adoption, Implementation, and Maintenance; TDF, Theoretical Domains Framework; TFA, Theoretical Framework of Acceptability; UTAUT, Unified Theory of Acceptance and Use of Technology.

## 4 Results

The initial database search returned 3,947 unique records after deduplication. Of these, 3,734 (95%) were excluded during title and abstract screening. Full-text publications of the remaining 213 papers were retrieved and assessed for eligibility. Of the 213 articles, 21 (10%) were included in the review. An additional 10 papers were identified from grey literature and citation searching, resulting in 31 papers included in this review.

### 4.1 Characteristics of included studies

Characteristics of included studies are presented in Table 4. Studies were published between 2007 and 2023, and the majority were conducted in The United States of America (USA; *n* = 18/31, 58%). Studies used primarily quantitative methods (n = 18/31, 58%), the remaining studies used qualitative (*n* = 4/31, 13%), or a mixed-methods approach (*n* = 9/31, 29%).

**Table 4:** Characteristics of included studies.

First author, year	Country of first author	Setting	Study design	Research method
Abdelmutti, 2019	Canada	Large cancer center	Implementation study	Quantitative
Andrews, 2011	USA	Public housing neighborhoods in two Southeastern US metropolitan communities	Cluster RCT	Mixed
Blok, 2019	USA	Four hospital units	Phased implementation study	Mixed
Craig, 2022	USA	Medical oncology outpatient clinics	Implementation study	Quantitative
Darker, 2022	Ireland	Socio-economically disadvantaged districts	Embedded qualitative design	Qualitative
De Los Reyes, 2023	USA	Homeless shelters in San Francisco	Single-arm, community-based uncontrolled trial and qualitative interviews	Mixed
Foley, 2023	USA	Community-based radiology facilities	Effectiveness-implementation hybrid type II cluster randomized trial	Quantitative
Fullerton, 2023	Ireland	Community- urban areas	Action research	Mixed
Gould, 2019	Australia	Aboriginal medical services in Australia	Pilot cluster randomized step-wedge trial	Mixed
Hayes, 2022	Ireland	Socio-economically disadvantaged districts	Pragmatic two-arm, parallel-group pilot cluster RCT	Quantitative
Hood-Medland, 2019	USA	University of California, Davis Health Systems (UCD), and the California Smokers’ Helpline	Prospective implementation study	Quantitative
Japuntich, 2022	USA	Community mental health centers	Qualitative interviews	Qualitative
Jones, 2020	UK	Hospital Trusts and local authority departments in the Northeast of England	Semi-structured interviews	Qualitative
Kim, 2012	USA	General Electric (GE) worksites	RCT	Mixed
Lachter, 2022	USA	American Indian Quitline in Minnesota	Multi-phase project (not a research study)	Qualitative
Landais, 2021	Netherlands	Amsterdam	Implementation study	Mixed
LeLaurin, 2020	USA	Outpatient clinics in the University of Florida Health System	Implementation study	Quantitative
Little, 2009	USA	Dental practice	Group-level RCT	Quantitative
Matthews, 2009	USA	Community	Development of a pilot study of a culturally targeted cessation intervention	Quantitative
Meijer, 2021	Netherlands	Municipalities that participate in the Dutch ministry of health program “healthy in the city”	Real-world study proof-of-concept implementation project	Mixed
Naughton, 2015	UK	Antenatal clinic	Single site service evaluation	Quantitative
Ni Mhurchu, 2019	New Zealand	Māori and Pasifika community settings	2-arm cluster RCT	Quantitative
Scheffers-van Schayck, 2021	Netherlands	Healthcare settings & mass media	Effectiveness-implementation hybrid trial	Quantitative
Shorey Fennell, 2023	USA	Healthcare center in Texas	Mixed methods evaluation	Mixed
Skelton, 2022	Australia	Clinic in inner-city hostel for homeless men	Pilot study with single group pre- and post-treatment follow-up design and embedded process evaluation	Quantitative
Smith, 2020	Canada	Acute-care community hospital located in a small rural municipality in northwestern Ontario, Canada	Stage 3 translational research implementation study	Quantitative
Tong, 2023	USA	University of California health systems	Not stated	Quantitative
Vidrine, 2013	USA	Family practice clinics in a single metropolitan area	Pair-matched, 2-treatment-arm, group randomized design with randomization at the clinic level	Quantitative
Wetter, 2007	USA	Telephone-based cessation counselling service within NCI Cancer Information Service	2-group RCT	Quantitative
Windsor, 2014	USA	Home-based	Quasi-experimental, non-randomized, matched comparison group design	Quantitative
Windsor, 2017	USA	Home-based	Comparative effectiveness evaluation	Quantitative

### 4.2 Interventions reported in the implementation of smoking cessation programs

A tabular description of interventions and characteristics are reported in Table 5. In total, 12 interventions were identified, including individual or group counselling, workbooks and self-help materials, and peer support. Most studies (*n* = 26/31, 84%) used a theoretical evidence base such as motivational interviewing (55), or clinical guidelines to develop and administer the intervention. Interventions were delivered by diverse providers including nurses, pharmacy staff, and peer supporters. Most studies (*n* = 27/31, 87%) reported delivering multicomponent interventions. Examples of interventions delivered included no-cost mono-or combination-pharmacotherapy reported in 12 studies (56–67). An additional four studies (68–71) reported provision of pharmacotherapy, but it was unclear whether there was a cost to participants. All studies offering free pharmacotherapy offered more than one intervention, with the majority including counselling (*n* = 11/12, 92%), alongside other intervention components. Three studies (58, 59, 72) included biofeedback in the form of exhaled carbon monoxide (CO) monitoring as a tool for education, and/or motivation for participants, in addition to verifying abstinence. One study (62) used CO monitoring to verify abstinence, and whilst biofeedback was not an intervention component, participants were offered the use of the CO monitor to track their own cessation progress if desired. Eleven studies used either CO monitoring (57, 63, 64, 66, 73, 74), or salivary or urinary cotinine (56, 60, 65, 69, 75) for the sole purpose of verifying abstinence, and not as an intervention component.

**Table 5:** Smoking cessation interventions.

First author, year		Abdelmutti, 2019	Andrews, 2011	Blok, 2019	Craig, 2022	Darker, 2022	De Los Reyes, 2023	Foley, 2023	Fullerton, 2015	Gould, 2019	Hayes, 2022	Hood-Medland, 2019	Japuntich, 2022	Jones, 2020	Kim, 2012	Lachter, 2022	Landais, 2021	LeLaurin, 2020	Little, 2009	Matthews, 2009	Meijer, 2021	Naughton, 2015	Ni Mhurchu, 2019	Scheffers-van Schayck, 2021	Shorey Fennell, 2023	Skelton, 2022	Smith, 2020	Tong, 2023	Vidrine, 2013	Wetter, 2007	Windsor, 2014	Windsor, 2017
Theoretical/ evidence base of intervention	CBT																	✓										✓				
	MI							✓	✓															✓		✓		✓		✓		
	3 A’s	✓																														
	Ask-Advise-Connect																✓								✓							
	5 A’s				✓			✓					✓																			
	Behavior change theory or model									✓							✓						✓									
	Social TMF					✓					✓																✓					
	Clinical guideline/ standards	✓	✓						✓					✓						✓	✓				✓				✓	✓	✓	✓
	Other			✓																		✓					✓					
	Not stated						✓					✓			✓	✓			✓													
Provider type	Nurses/ midwives	✓		✓	✓					✓				✓	✓												✓		✓		✓	✓
	Medical doctors	✓			✓					✓		✓								✓												
	Pharmacy staff	✓				✓	✓	✓	✓																							
	Other health/ medical staff	✓			✓			✓		✓	✓									✓					✓	✓			✓			
	Dentists/ dental staff	✓																	✓													
	Counsellors																		✓	✓				✓				✓		✓		
	Social workers												✓																		✓	✓
	Other mental health professionals												✓																			
	Tobacco treatment professionals		✓					✓	✓							✓	✓	✓														
	Other professional staff	✓					✓			✓	✓		✓	✓	✓												✓					
	Peer/ community supporters		✓			✓			✓		✓																					
	N/A (e-Health)																				✓	✓	✓									
Delivery mode	Built into health/ social services	✓		✓				✓		✓		✓																				
	In-person		✓	✓	✓	✓		✓	✓	✓	✓			✓	✓		✓		✓	✓						✓	✓		✓		✓	✓
	Telephone						✓		✓			✓	✓			✓	✓		✓					✓		✓	✓	✓	✓	✓		
	SMS			✓					✓							✓						✓										
	Digital/ virtual	✓								✓						✓		✓			✓		✓								✓	✓
	Mailed		✓																									✓		✓		
Intervention component	Individual counselling^a^		✓				✓		✓	✓				✓		✓	✓	✓	✓					✓		✓	✓	✓	✓	✓	✓	✓
	Group counselling^a^		✓			✓			✓		✓						✓			✓							✓					
	Peer support		✓			✓					✓																					
	Brief advice				✓			✓						✓				✓	✓										✓			
	Referral to Quitline/counselling	✓			✓							✓	✓	✓				✓	✓						✓			✓	✓			
	Automated program^b^	✓		✓																	✓	✓	✓									
	Non-automated program^c^								✓																							
	Integrated in EHR/ between services	✓		✓	✓							✓													✓		✓	✓		✓	✓	✓
	Workbooks/printed materials		✓	✓		✓		✓		✓				✓			✓			✓			✓	✓				✓		✓	✓	✓
	Financial incentives														✓																	
	Biofeedback^d^								✓	✓				✓				✓^e^														
	No cost pharmacotherapy		✓		?	✓	✓	?	✓	✓	✓					✓	✓	✓		✓					✓	✓	✓		?			
Pharmaco-therapy types	NRT		✓		✓	✓	✓		✓	✓	✓					✓	✓	✓		✓					✓	✓	✓		✓			
	Varenicline				✓																					✓						
	Bupropion				✓																											
	Pharmacotherapy not specified							✓																								

? Access to pharmacotherapy provided, unclear whether free of charge. 3 A’s, Ask Advise Act; 5 A’s, Ask, Advise, Assess, Assist, and Arrange; CBT, Cognitive behavior therapy; CO, Exhaled Carbon Monoxide; EHR, Electronic health record; MI, Motivational interviewing; N/A, Not applicable; NRT, Nicotine replacement therapy; SMS, Short message service; Social TMF, Social theory, model, or framework.

a Counselling methods including psycho-education.

b Automated programs or systems, e.g., automated SMS sent from message bank, mobile apps.

c Non-automated programs or systems, e.g., SMS chat/ online chat.

d Biofeedback as an intervention component as opposed to biochemical validation of abstinence.

e Participants free to use CO monitor for personal tracking if desired. Biofeedback not strictly an intervention component.

**Figure 3:**
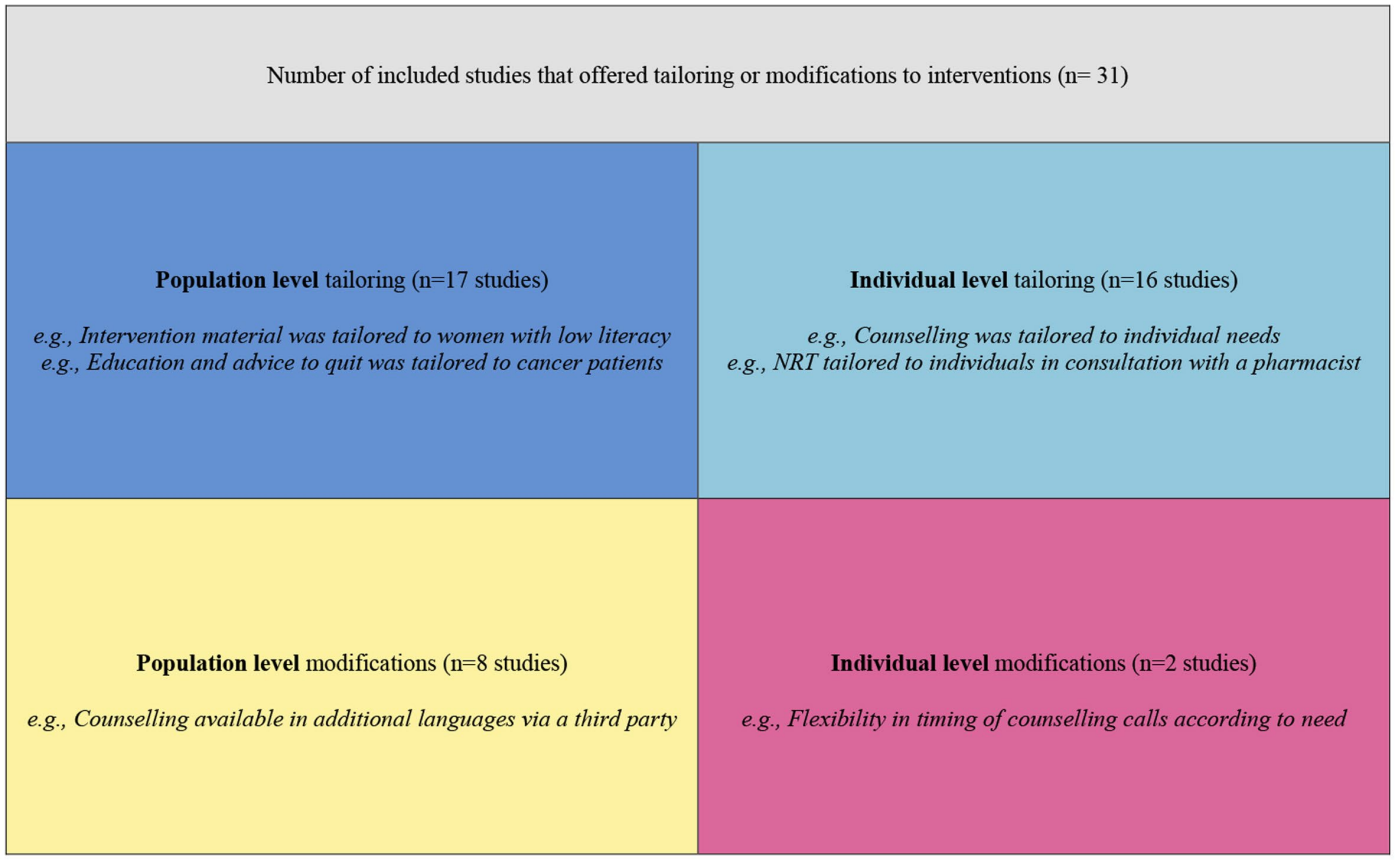
Tailoring and modifications.

Tailoring and/or modifications were a common component of interventions across included studies (*n* = 28/31, 90%; Figure 3). We defined tailoring as service provision that takes into consideration the characteristics and needs of the people that the service is being delivered to at individual and population levels (76). Whereas modifications were broadly defined as alterations or additions to the design or delivery of interventions, whether intentional or inadvertent (77). A summary of tailoring and modifications is provided in Table 6. Seventeen studies employed tailoring of interventions at the population level, including developing culturally-tailored interventions for the intended populations (59, 61, 63, 78, 79), gender specific interventions for women (56, 58, 60, 72–74), tailored to the needs and characteristics of the intended populations (65, 66, 75, 80) and for specific patient groups (62, 81). Sixteen studies tailored interventions at the individual-level (57, 58, 60, 65–67, 70, 72–74, 78, 80, 82–85). For example, one study (57) engaged pharmacists to assess, and provide nicotine replacement therapy (NRT) according to each participant’s needs. Another study (84) included several dimensions of tailoring to pregnant participants, including messages via short messaging service (SMS) addressing participants by their first name with information about fetal development and pregnancy relevant to gestation. Messaging content was tailored according to participants’ responses, with the additional capacity to send an SMS to request more or less messages from the program. Participants who did not respond to messages or provide their details, would receive generic, non-tailored SMS. Another common form of tailoring among included studies was in the form of individual counselling tailored to the individual (58, 66, 67, 70, 73, 74, 80, 85). Notwithstanding tailoring, eight studies made modifications to the intervention for the intended population (67, 69, 71, 81, 84–87). Modifications most commonly consisted of counselling and written information made available in different languages (67, 71, 81, 85). Two studies made individual-level modifications which included offering flexibility with the timing of telephone calls with participants (71), or adding smokers in the participant’s household to the referral intervention where previously only individual smoking participants were included (86).

**Table 6:** Tailoring and modifications.

First author, year	Population level	Individual level
Tailoring		
Abdelmutti, 2019	Education and advice to quit tailored to cancer patients	N/A
Andrews, 2011	Cessation handbook written at 3rd–4th grade reading level, designed to accompany each group session. Handbook developed with multiple revisions during formative work based on focus group and process evaluation measures	Providers incorporated their own language and cultural style. Providers were able to share testimonials and personal experiences, offer to pray with individuals, share bible passages, cultural poems and inspirational themes
Darker, 2022	Intervention tailored to socio-economic disadvantaged women	N/A
De Los Reyes, 2023	N/A	NRT tailored according to individual’s needs in consultation with pharmacist
Fullerton, 2015	Program tailored to the needs of women in Ireland	Counselling tailored to individuals
Gould, 2019	Culturally specific intervention	N/A
Hayes, 2022	Intervention material was tailored to women with low literacy	Sessions 7–12 tailored to the preferences and needs of each group
Jones, 2020	Intervention was designed for pregnant smokers	Additional intervention was delivered by midwives for smokers who did not engage with Stop Smoking Services
Kim, 2012	Organizational- and individual-level barriers and facilitators were explored, and recruitment strategies were tailored to worksites	N/A
Lachter, 2022	Culturally tailored intervention	N/A
Landais, 2021	Intervention adapted to the specific needs of lower SES smokers, including modelling, practical learning, reinforcement, and feedback. Verbal and written language were in Dutch at an intermediate proficiency level	Counselling tailored to individuals
LeLaurin, 2020	Tailored to cancer patients	N/A
Little, 2009	N/A	Dental staff and counsellors provided personalized advice, and explored personal motivations and barriers for quitting with patients
Matthews, 2009	Culturally tailored intervention	N/A
Meijer, 2021	N/A	Motivational messages tailored to participant responses and addressed participants by App username
Naughton, 2015	N/A	Messages addressed participants by first name and tailored to participant responses. Additional SMS sent if quit date provided, gestation-tailored baby development information, additional smoking in pregnancy risk information if user responded to prompt SMS. Non-tailored support if tailoring questions not answered
Ni Mhurchu, 2019	Culturally tailored for Māori/ Pasifika people	Mobile app intervention pre-programmed with a list of generic behavior change goals but fully customizable to user goals and progress, specific to Māori or Pasifika people
Scheffers-van Schayck, 2021	Printed material relevant to parents who want to quit smoking	Phone counselling tailored to individuals
Shorey Fennell, 2023	N/A	Counselling tailored to individuals
Smith, 2020	N/A	Counselling tailored to individuals
Tong, 2023	N/A	Counselling tailored to individuals
Wetter, 2007	Counselling culturally tailored for Hispanic culture and delivered in Spanish	N/A
Windsor, 2014	Video intervention tailored to pregnant women	Counselling tailored to individuals
Windsor, 2017	Video intervention tailored to pregnant women	Counselling tailored to individuals
Modifications		
Abdelmutti, 2019	Written and verbal education available in English, Chinese, Portuguese, Russian, Spanish, and Vietnamese	N/A
Blok, 2019	Messages not appropriate for use in the inpatient setting (i.e., “take a walk outside”) were not included in the posters	N/A
Foley, 2023	Sites were given the option to personalize their toolkits	N/A
Hood-Medland, 2019	Referrals were added to inpatient discharge orders	Inpatient discharge orders were modified to include referrals for household smokers
Naughton, 2015	Intervention adapted for use in routine antenatal care	N/A
Shorey Fennell, 2023	Counselling offered in English and Spanish, and at least 15 other languages via a third party	N/A
Tong, 2023	Counselling available in English, Spanish, Cantonese, Mandarin, Vietnamese, or Korean	N/A
Vidrine, 2013	Counselling available in English and Spanish, at least 15 additional languages available via third party	Timing of calls was flexible, and modified according to need

N/A, Not applicable; NRT, Nicotine replacement therapy; SES, Socioeconomic status; SMS, Short message service.

### 4.3 Implementation theories, models, and frameworks reported by smoking cessation programs

A summary of implementation TMFs, or other approaches to guide implementation is provided in Table 3. We identified substantial heterogeneity in TMFs across studies. Of the included studies, 26 out of 31 used 19 distinct implementation TMFs to prospectively guide and evaluate implementation. Whilst there was heterogeneity in the use of TMFs across studies overall, this was less apparent when observed by country. The Reach Effectiveness Adoption Implementation Maintenance (RE-AIM) Framework (25) was the most commonly applied TMF, used in seven studies (67, 68, 71, 75, 79, 86, 87), all based in the USA. Studies that took place in the USA (*n* = 18) applied 10 distinct TMFs and/ or community/ co-design approach. Only one study applied more than one TMF or approach (87). In contrast, studies taking place outside of the USA (*n* = 13/31, 42%) applied 15 distinct TMFs and/ or community/ co-design approaches. Five of these studies used more than one TMF or approach (59, 60, 72, 78, 83). For example, the Theoretical Domains Framework (88) was applied in three studies taking place in Australia, New Zealand, and the United Kingdom (59, 72, 78); these studies also applied co-design with First Nations health services and communities or other TMF.

Examples of the application of TMFs included one study (59) which used both the Theoretical Domains Framework (88) and the Behavior Change Wheel (28) to inform the collaborative design of an intervention with a First Nations community. The frameworks supported the development of an intervention for health providers to the community, which was subsequently implemented. Another study (69) used an effectiveness-implementation hybrid design to support implementation over a number of stages, starting prior to commencement of the research by evaluating the suitability of potential sites to implement the intervention. Included studies that did not use a formal TMF (*n* = 5/31, 16%) included real-world, implementation projects or used community engagement in the implementation effort (58, 61, 62, 70, 84).

Nineteen studies (56, 57, 59, 63–67, 71–75, 81–83, 85, 86, 89) provided evidence of how the chosen TMF aligned with planning, implementation, or evaluation (90). Examples of this included one study (66) which used intervention mapping (91) through a multi-phased adaptation process. This process included an exploration, preparation, and implementation phase. A detailed description of the processes within each phase of the implementation aligned with the processes described in intervention mapping (91). Another study (85) provided descriptions of the implementation strategies used in the implementation process and how they aligned to the discrete ERIC strategy domains (53), in addition to post-implementation activities.

### 4.4 Implementation strategies reported by smoking cessation programs

Diverse approaches to implementation were reported across studies. Of the included studies, one paper (85) used the refined ERIC strategy domains (53) to guide implementation. The remaining studies did not use ERIC (53) but we evaluated what was reported against the definitions provided in the ERIC Discrete Implementation Strategy Compilation (92). Findings are summarized in Table 7. Of the nine domains, “use evaluative and iterative strategies” (*n* = 24/31, 77%), “adapt and tailor to context” (*n* = 19/31, 61%), and “train and educate stakeholders” (*n* = 22/31, 71%) were the most commonly applied strategy domains identified. We did not identify any implementation strategies that could not be mapped to the ERIC compilation (53).

**Table 7:** Implementation strategies mapped to ERIC domains.

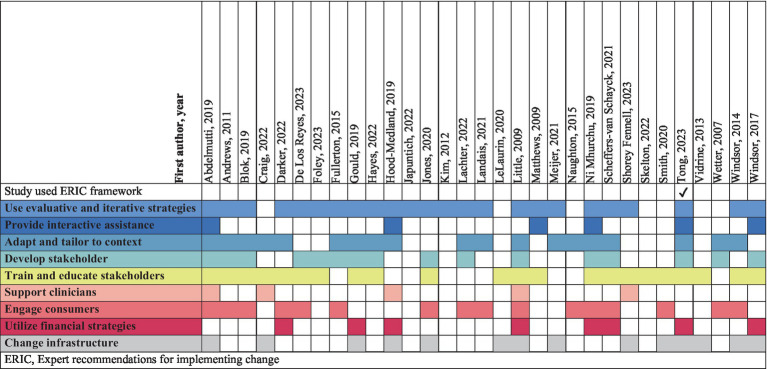

ERIC, Expert recommendations for implementing change.

An example of strategies that fell within the ERIC domain “use evaluative and iterative strategies” was identified in one study (74) that developed a “Process Evaluation Model,” to measure adoption and implementation through a “Program Implementation Index (PII).” Individuals involved in various aspects of the implementation effort provided or received training in implementing the program intervention. Performance data were collected throughout study implementation and reviewed quarterly against the PII performance metric. A PII score of 80% or above was considered the standard for adoption. Providers with a PII over 90% were involved in reviewing the Process Evaluation Model results and provided advice on program policies for subsequent phases. After implementation of the new policies, the PII of providers was reviewed again. Whilst providers achieving a PII of 80% or above were sent congratulatory letters, those with a PII of 79% or less would be entered into a quality improvement plan with their supervisor and required re-training.

An example of strategies that fell within the ERIC domain “Adapt and tailor to context” was demonstrated in one study (81) which used a phased approach to implementation. In early phases, professionals were engaged to develop and integrate the intervention into existing digital infrastructure. Piloting was performed to identify how to adapt the intervention to the existing infrastructure, and audits took place to identify issues and needs at each site, to integrate the intervention into the clinical systems.

Another study (58) engaged strategies that fell within the ERIC domain “train and educate stakeholders.” This study engaged a “train the trainer” program, modifying an existing training program to align with the study’s aims. Training was performed by coordinators who developed and distributed materials to community facilitators who were instructed on how to deliver smoking cessation counselling to the intended population. Following the initial two and a half days of training, community facilitators received ongoing mentoring and support through scheduled phone calls or SMS. Community facilitators met in-person to evaluate and plan ongoing program delivery, and provide feedback to the coordinators about the training, and training procedures and materials for future programs.

### 4.5 Other findings

Tailoring and modifications of interventions at the individual and population levels was employed in 28 out of 31 studies (Figure 3). In the process of evaluating tailoring, we identified three studies (59, 61, 78) in which tailoring was distinct. One study (78) used an implementation TMF (27) alongside behavior change theory (93) and a community participatory research approach. The researchers in this study formed an academic-community partnership established on participation and protection of the First Nations people, drawn from the principles of the founding Treaty of the country in which the study took place (94). The Treaty principles formed the basis of culturally informed community engagement, exploration, planning, feedback, design, iterative development, and piloting throughout the implementation. This study information was not included in the paper, but was detailed on the study website (95). Another study (59) was implemented in a similar fashion, using TMFs (27, 96) to guide culturally sensitive implementation according to First Nations ethics guidelines (97). The researchers partnered with a Stakeholder and Consumer Advisory Panel that included community members and service providers, to collaboratively guide First Nations-specific implementation for the communities involved in the project. Collaboration, cultural sensitivity, and First Nations ownership were emphasized through every stage of the project implementation. This information was outlined in the study protocol and related documents (98–100). A third study (61) did not use an implementation TMF, but used a community engagement framework (101) to guide the participatory approach. Partnership, and shared power and responsibility were emphasized through equitable inclusion of the goals and perspectives of the community, shared purpose, and mutual benefit. The community was engaged throughout project development, training of providers, implementation and monitoring. Community partners provided cultural guidance and designed and lead the community collaboration process. Roles and responsibilities were designated to each of the project partners according to their expertise.

## 5 Discussion

To our knowledge, this is the first scoping review to evaluate the implementation of smoking cessation interventions alongside implementation theories, models, and frameworks, concurrently mapping implementation strategies to ERIC (53). We identified relevant studies across a range of designs and methods, participant groups, clinical and community settings, and geographic locations. Studies included a broad range of intervention types, delivery modes, providers, and theoretical evidence bases to inform intervention development. Evidence bases most commonly included motivational interviewing (55), and clinical guidelines or standards. There was substantial heterogeneity in TMFs reported across studies. Implementation strategies identified covered all nine ERIC domains, most of which were individual-level strategies (53). Our finding that no strategies fell outside of ERIC is somewhat surprising considering ERIC was compiled and refined in clinical, and mental health, rather than the community-based contexts that were the focus of this review (102).

This review summarized interventions used during the implementation of smoking cessation programs. Our results highlight the numerous intervention types, methods of delivery, providers, and theoretical foundations for implementing smoking cessation interventions in the community. We identified multicomponent interventions tailored to the intended populations, and/or individuals reported in most studies (*n* = 27/31). Multicomponent, tailored interventions have been shown to be more effective than single, less complex interventions for smoking cessation, particularly for vulnerable, at-risk, and culturally diverse groups (103–105). Furthermore, we identified peer counsellors and community supporters among intervention providers, in addition to peer support as an intervention component. Our findings corroborate with previous research emphasizing the importance of social support alongside intensive, multicomponent interventions (103), However, we did not evaluate the duration of interventions in included studies, which has been identified as an important element of cessation support (103).

We evaluated implementation theories, models and frameworks, limiting the inclusion criteria to studies using an implementation TMF, or other identifiable implementation components (23). Thus, most (*n* = 26/31) of the included studies used at least one TMF. In two studies that did not use a TMF (59, 61), and one study that did use a TMF (78), community participation and engagement was a strong component of implementation (58). Whilst there are numerous community participatory frameworks for implementation (106–110), a number of studies using community participation to guide the implementation without specifying TMF use have been reported previously in a review (111). There is current debate in the literature suggesting that “common sense” in the form of local knowledge, which could be garnered through community participation, could replace TMFs to guide implementation (32). Whereas others contend that conscientious use of TMFs can support the ability to plan, guide, conduct, and evaluate implementation (29). Furthermore, that TMFs can improve the generalizability and translation of findings through shared terminology and knowledge (29). This suggests that whilst community participation may be considered an effective standalone approach to implementation, judicious application of TMFs may support successful implementation, and translation of theory to practice. Translation to practice is particularly important in countries such as Australia, Canada, and New Zealand, where the First Nations people have considerably higher smoking prevalence due to the ongoing effects of colonization (112). As such, TMF selection should take into consideration the strengths and diversity, in addition to the needs and preferences of these populations for future implementation (113). Furthermore, Australia covers an expansive geographic area, with diverse communities spanning from urban, to very remote and varying access to health services (114). Such contextual factors should be considered when selecting a TMF, to ensure that implementation extends to populations that historically may have been excluded from implementation projects (115, 116).

We excluded studies that did not use a TMF, or other identifiable elements of implementation. Consequently, 129 studies reporting on implementation of smoking cessation programs, including “real-world” studies, were excluded from this review (Figure 2). Reasons for underutilization of TMFs may be due to a number of factors, including difficulty selecting from the overwhelming number of TMFs available, hesitancy in applying a TMF due to lack of knowledge or experience, or confusion due to inaccurate and inconsistent definitions related to implementation terminology (30, 117, 118). Whilst a TMF is not prerequisite for implementation, as demonstrated in a number of included studies (58, 61, 62, 70, 84), there are a number of possible implications to not applying TMFs to an implementation effort. For example, potential benefits for planning, delivery, evaluation, and reporting the implementation of smoking cessation programs may not be realised where a TMF is not applied (29, 119). When conducting full-text screening, we encountered a challenge in differentiating between implementation strategies and intervention components in some studies lacking a TMF, due to unclear terminology and definitions (23), and a lack of clear and methodical structure for implementation processes (120). Whilst smoking cessation interventions have been extensively researched (121), implementing effective interventions into routine practice is vital to combat the rising trajectory of morbidity and mortality related to tobacco smoking (6). Following the advice of others, we suggest taking a purposeful approach, applying the appropriate knowledge and tools that align with the studies purpose and goals to guide and support implementation (117, 119). The process of selecting appropriate TMFs for the target population, study purpose, or context, could be supported by use of checklists and tools (122). Deliberate and appropriate use of TMFs may support the transferability of implementation to other interventions and contexts (29).

We mapped implementation strategies in included studies across the strategy clusters detailed in ERIC, and did not identify any implementation strategies that could not be mapped to ERIC (53). Whilst strategies that fall outside of ERIC have been identified in other studies (123, 124), our findings suggest that ERIC (53) could be an appropriate framework for implementing smoking cessation interventions. We identified diverse approaches to implementation within the ERIC strategy domains in included studies. Individual-level strategies, within the domains of ‘use evaluative and iterative strategies’, ‘adapt and tailor to context’, ‘development of stakeholder interrelationships’, ‘train and educate stakeholders’, and ‘engage consumers’ were commonly applied across included studies. Whereas we noted less common application of service-level strategies within the domains of ‘provide interactive assistance’, ‘utilize financial strategies’, and ‘change infrastructure’. This finding may be due to use of TMFs other than ERIC to guide implementation in all but one study (85), or approaches to implementation without use of a TMF. Alternatively, these systems-level strategies may have been considered incompatible with the aims or scope of the included studies, nevertheless these strategies are vital to translating evidence to practice (125). We suggest that future studies implementing smoking cessation interventions should consider incorporating strategies across ERIC domains to support tailoring of implementation efforts, and translation to practice (126).

A novel finding in this review was the cultural tailoring applied to the process of implementation in three studies (59, 61, 78). Cultural tailoring includes modifications to study procedures and interventions in response to the cultural needs of the population (127), and has been established as an important component of smoking cessation and health services (104, 128, 129). Cultural tailoring is not limited to race and ethnicity, and refers to the shared characteristics that shape the attitudes and behaviors of a population through their interactions with their environment (130). In the three studies (59, 61, 78) that developed culturally tailored interventions, we identified further evidence that the implementation was culturally tailored through purposefully embedding Treaty principles (94), First Nations Ethics and Guidelines (97), and culturally guided application of a community engagement framework (101) to the implementation process. The collaboration between the researchers and communities, development of partnerships, shared decision making, and culturally guided, community-driven intervention and implementation planning, development, delivery, and evaluation aligns with a culture-centered approach (131). Community participation and ownership of the projects was emphasized throughout the implementation of the aforementioned studies (59, 61, 78). These approaches have been previously described using a co-creation lens, which encompasses principles of equity, reflexivity, reciprocity and mutuality, transformation and personalization, and relationship facilitation (132). Emphasis on health equity through genuine community engagement and partnership, shared goals and power, centered on the needs and culture of the community has been identified as critical to progressing the field of implementation science (133). Whilst the culture-centered approach and co-creation have been previously described (131, 134), employing and identifying such strategies in research may be hampered due to confusing terminology, a lack of guidance for design and implementation, and a need for more research on applying frameworks to planning and implementation (134–136). Given the effectiveness of culturally tailoring smoking cessation interventions (104, 137–139), we believe that tailoring the implementation process according to culturally specific guidelines and principles (94, 97) (hereafter referred to as cultural TMFs) has the potential to improve the implementation of smoking cessation programs for intended populations. This could be achieved firstly through advancing terminology and knowledge surrounding the culture-centered approach and the co-creation lens (131, 132). Secondly, by harmonizing approaches described in the aforementioned models, and finally, through improved understanding of how to select and apply appropriate cultural TMFs to implementation projects.

## 6 Strengths and limitations

To the best of our knowledge, there are currently no comprehensive reviews mapping smoking cessation interventions, implementation theories, models and frameworks, and implementation strategies. This review contributes substantial knowledge to further the future implementation of smoking cessation interventions in community settings. Nevertheless, there are a number of limitations to this review. Five papers were excluded during full-text screening due to being non-English, additionally we did not include abstracts for which there was no full-text available. We limited the study setting to the community, therefore studies taking place exclusively in inpatient hospital settings were excluded thus limiting the generalizability of the results to non-community based contexts and providers. Whilst we provided a comprehensive report of smoking cessation interventions, TMFs, and implementation strategies, we did not evaluate the intensity and duration of interventions, their effectiveness for smoking cessation outcomes, nor implementation success. Furthermore, we did not evaluate the quality of implementation TMFs for smoking cessation interventions within included studies. Future systematic reviews could build on the findings of this review to evaluate the comparative effectiveness of theory-and non-theory-based implementation strategies for smoking cessation interventions. We mapped implementation strategies to ERIC, however, we acknowledge other taxonomies for implementation strategies exist and could be considered for mapping implementation strategies for smoking cessation programs. Finally, this review did not evaluate de-implementation, which is often required alongside implementation efforts and should be considered in future reviews.

## 7 Conclusion

This scoping review identified interventions, TMFs and other approaches, and implementation strategies for smoking cessation programs. We identified broad use of numerous, multi-component, and tailored interventions, by diverse providers, for smoking cessation programs, emphasizing the strategies by which cessation may be supported. These strategies included non-TMF approaches including co-design and community engagement. Culturally tailored implementation emerged as a distinct implementation strategy in three studies. Harmonizing strategies using a culture-centered approach and co-creation lens alongside relevant cultural TMFs could be considered as a means to improve implementation for intended populations, and thus public health.
