# Supplementary material for: Evaluating the implementation of adult smoking cessation programs in community settings: a scoping review
Source: Front Public Health. 2025 Mar 28;12:1495151. doi: 10.3389/fpubh.2024.1495151 (PMC11988889; doi:10.3389/fpubh.2024.1495151)
Supplement: Supplementary file 1 [file Table_1.docx]

Supplementary Table 1 Database search terms

| **MEDLINE (EBSCOhost)** | (MH ("Smoking Cessation" OR "Tobacco Use Cessation")) OR (TI (smok* OR tobacco OR nicotine OR cigarette) N3 (quit* OR cease* OR cessat* OR reduc* OR prevent OR stop* OR "cut down" OR abstain* OR abstin* OR maint*) OR AB (smok* OR tobacco OR nicotine OR cigarette) N3 (quit* OR cease* OR cessat* OR reduc* OR prevent OR stop* OR "cut down" OR abstain* OR abstin* OR maint*)) |
| --- | --- |
|  | AND |
|  | (MH ("Counseling+" OR "Telephone+" OR "Complementary Therapies+" OR "Internet-Based Intervention")) OR (TI (Quitline OR "quit line") OR AB (Quitline OR "quit line")) OR (TI ((Phone OR Telephone OR Web* OR Mobile* OR Technolog* OR Peer* OR communit* OR community-based OR community-led OR Smartphone OR Telehealth) N3 (counsel#ing OR service OR intervention OR support OR program* OR app*)) OR AB ((Phone OR Telephone OR Web* OR Mobile* OR Technolog* OR Peer* OR communit* OR community-based OR community-led OR Smartphone OR Telehealth) N3 (counsel#ing OR intervention OR service OR support OR program* OR app*))) |
|  | AND |
|  | (MH ("Implementation Science" OR "Program Evaluation")) OR (TI (implement* OR evaluat*) OR AB (implement* OR evaluat*)) |
| **Cochrane CENTRAL trials Library** | (MeSH descriptor: [Smoking Cessation] explode all trees) OR (MeSH descriptor: [Tobacco Use Cessation] explode all trees) OR (((smok* OR tobacco OR nicotine OR cigarette) NEAR/3 (quit* OR cease* OR cessat* OR reduc* OR prevent OR stop* OR "cut down" OR abstain* OR abstin* OR maint*)):ti OR ((smok* OR tobacco OR nicotine OR cigarette) NEAR/3 (quit* OR cease* OR cessat* OR reduc* OR prevent OR stop* OR "cut down" OR abstain* OR abstin* OR maint*)):ab) |
|  | AND |
|  | (MeSH descriptor: [Counseling] explode all trees) OR (MeSH descriptor: [Telephone] explode all trees) OR (MeSH descriptor: [Complementary Therapies] explode all trees) OR (MeSH descriptor: [Internet Based Intervention] explode all trees) OR (Quitline OR "Quit line"):ti OR (Quitline OR "Quit line"):ab) OR (((Phone OR Telephone OR Web* OR Mobile* OR Technolog* OR Peer* OR communit* OR community-based OR community-led OR Smartphone OR Telehealth) NEAR/3 (counsel#ing OR service OR intervention OR support OR program* OR app*)):ti OR ((Phone OR Telephone OR Web* OR Mobile* OR Technolog* OR Peer* OR communit* OR community-based OR community-led OR Smartphone OR Telehealth) NEAR/3 (counsel#ing OR service OR intervention OR support OR program* OR app*)):ab) |
|  | AND |
|  | (MeSH descriptor: [Health Plan Implementation] explode all trees) OR (((implement* OR evaluat*)):ti OR ((implement* OR evaluat*)):ab) |
| **Web Of Science Core Collection (Clarivate)** | (TI=((smok* OR tobacco OR nicotine OR cigarette) NEAR/3 (quit* OR cease* OR cessat* OR reduc* OR prevent OR stop* OR "cut down" OR abstain* OR abstin* OR maint*))) OR (AB=((smok* OR tobacco OR nicotine OR cigarette) NEAR/3 (quit* OR cease* OR cessat* OR reduc* OR prevent OR stop* OR "cut down" OR abstain* OR abstin* OR maint*))) |
|  | AND |
|  | ((TI=(Quitline OR "quit line")) OR (AB=(Quitline OR "quit line"))) OR ((TI=((Phone OR Telephone OR Web* OR Mobile* OR Technolog* OR Peer* OR communit* OR community-based OR community-led OR Smartphone OR Telehealth) NEAR/3 (counseling OR service OR intervention OR support OR program* OR app*))) OR AB=((Phone OR Telephone OR Web* OR Mobile* OR Technolog* OR Peer* OR communit* OR community-based OR community-led OR Smartphone OR Telehealth) NEAR/3 (counseling OR service OR intervention OR support OR program* OR app*))) |
|  | AND |
|  | (TI=((implement* OR evaluat*))) OR AB=((implement* OR evaluat*)) |
| **Embase (Elsevier)** | ('smoking cessation'/exp OR 'smoking cessation program'/exp) OR ((((smok* OR tobacco OR nicotine OR cigarette) NEAR/3 (quit* OR cease* OR cessat* OR reduc* OR prevent OR stop* OR 'cut down' OR abstain* OR abstin* OR maint*)):ti) OR (((smok* OR tobacco OR nicotine OR cigarette) NEAR/3 (quit* OR cease* OR cessat* OR reduc* OR prevent OR stop* OR 'cut down' OR abstain* OR abstin* OR maint*)):ab)) |
|  | AND |
|  | ('patient counseling'/exp OR 'mobile phone'/exp OR 'telephone'/exp OR 'alternative medicine'/exp OR 'web-based intervention'/exp) OR (((quitline:ti OR 'quit line'):ti) OR ((quitline:ab OR 'quit line'):ab)) OR ((((phone OR telephone OR web* OR mobile* OR technolog* OR peer* OR communit* OR 'community based' OR 'community led' OR smartphone OR telehealth) NEAR/3 (counselling OR counseling OR service OR intervention OR support OR program* OR app*)):ab) OR (((phone OR telephone OR web* OR mobile* OR technolog* OR peer* OR communit* OR 'community based' OR 'community led' OR smartphone OR telehealth) NEAR/3 (counselling OR counseling OR service OR intervention OR support OR program* OR app*)):ti)) |
|  | AND |
|  | ('implementation science'/exp) OR (((implement* OR evaluat*):ti) OR ((implement* OR evaluat*):ab)) |
| **Google Advanced**  **Google Scholar** | Smoke OR smoking OR tobacco OR nicotine OR cigarette OR quit OR cease OR cessation OR abstain OR abstinence |
|  | AND |
|  | Counseling OR phone OR “alternative medicine” OR “web-based intervention” OR quitline OR “quit line” OR “community based” OR telehealth |
|  | AND |
|  | Implementation |

Supplementary Table 2 Data extraction template

| **Extraction Category** | **Data** | **Comments** |
| --- | --- | --- |
| First author, year |  |  |
| Country (first author) |  |  |
| Year study took place |  |  |
| Setting |  |  |
| Study design |  |  |
| Research method |  |  |
| Sample size |  |  |
| Study title |  |  |
| Participant population |  |  |
| Intervention providers |  |  |
| Theoretical/ evidence base of intervention |  |  |
| Intervention description |  |  |
| Mechanism of delivery (e.g., group sessions in-person) |  |  |
| Number, frequency, intensity, duration of intervention |  |  |
| Tailoring and modifications (description and whether individual or population level) |  |  |
| NRT or pharmacotherapy (type, cost, amount, duration, any other details) |  |  |
| Incentives offered |  |  |
| Abstinence biochemically verified (yes/ no, state whether CO or salivary cotinine) |  |  |
| Implementation TMF |  |  |
| Implementation strategies |  |  |
| Implementation outcomes |  |  |
| Contextual factors or barriers/ facilitators to implementation |  |  |
| Comments |  |  |
| URL or access intervention materials |  |  |
| Reference |  |  |
